# Association of neutrophil count with lung cancer risk and prognosis: A Mendelian randomization and NHANES analysis

**DOI:** 10.1097/MD.0000000000047510

**Published:** 2026-01-30

**Authors:** Chunpeng Meng, Zhifeng Zheng, Jianhua Li, Yong Liu, Qinghai Meng, Huan Wang, Xiaoren Hu, Binbin Lan, Rui Liu, Hanqing Li, Shuai Sun

**Affiliations:** a Department of Thoracic Surgery, The Seventh Clinical College of Shanxi Medical University, Linfen People’s Hospital, Linfen City, Shanxi, China.

**Keywords:** GWAS, lung cancer, Mendelian randomization, National Health and Nutrition Examination Survey, neutrophil count

## Abstract

Neutrophil count is closely related to the development of various tumors. This study aimed to assess the impact of neutrophil count on lung cancer incidence and survival. Genetic variants associated with neutrophil count were selected as instrumental variables from genome-wide association study data, and the causal relationship between the neutrophil count and lung cancer risk was assessed using Mendelian randomization (MR) analysis. Separately, survival analysis was used to assess the effect of neutrophil count on survival in adults with lung cancer using data from the National Health and Nutrition Examination Survey database. Eleven single-nucleotide polymorphisms were used as instrumental variables to assess the effect of neutrophil count. MR analysis showed that elevated neutrophil counts were associated with a significantly reduced risk of lung cancer (odds ratio: 0.77, 95% confidence interval [CI]: 0.62–0.95, *P* = .017) and lung adenocarcinoma (odds ratio: 0.63, 95% CI: 0.45–0.87, *P* = .005). Conversely, Kaplan–Meier survival analysis showed that a low neutrophil count was associated with significantly longer overall survival in individuals with lung cancer than a high neutrophil count (hazard ratio [HR]: 0.53, 95% CI: 0.31–0.90, *P* = .017). Cox regression revealed a significant interaction between a high neutrophil count and age (*P* = .003); the effect of a high neutrophil count on prognosis was significant in adults aged under 65 years (HR: 0.21, 95% CI: 0.10–0.47, *P* < .001) but a high neutrophil count was not associated with prognosis in adults aged 65 years and older (HR: 1.17, 95% CI: 0.57–2.39, *P* = .671). MR analysis revealed that neutrophil count plays a causal role in protecting against lung cancer, whereas survival analysis revealed that, in individuals with lung cancer, a higher neutrophil count is associated with a worse prognosis. The specific pathogenic mechanisms by which neutrophils affect lung cancer development and prognosis warrant further study.

## 1. Introduction

Lung cancer is a leading cause of cancer death worldwide, with a high incidence and mortality rate. Despite progress in diagnosis and treatment, its prognosis remains poor, with 5-year survival rates ranging between 4% and 17%, depending on the stage and region.^[1]^ New biomarkers are urgently required to enhance early diagnosis, personalize treatment for patients with lung cancer, and assist in risk and prognosis assessment.^[2,3]^

Neutrophils are crucial for inflammation and the immune response. Recent research has highlighted the significant role of inflammation in tumor initiation, progression, and metastasis,^[4]^ suggesting that neutrophils affect the pathophysiological processes of lung cancer by modulating the tumor microenvironment (TME).^[5]^ Clinical evidence has demonstrated a link between elevated neutrophil counts and poor prognosis in various tumors; however, the specific association between neutrophil count, lung cancer incidence, and survival of individuals with lung cancer requires further investigation.^[6–9]^

In traditional epidemiological studies, confounding factors and reverse causality often affect the accuracy of the assessment of causal effect. Mendelian randomization (MR) analysis uses genetic variation as an instrumental variable (IV) to infer causal relationships between exposure factors and outcomes from a genetic perspective, effectively reducing confounding.^[10,11]^ MR has been increasingly applied to explore the causal role of inflammation in the development of cancer. For example, Zhu et al^[12]^ conducted a pan-cancer MR analysis of circulating C-reactive protein and found no significant associations between genetically predicted C-reactive protein and cancer risk, despite observational associations. Li et al^[13]^ and Wu et al^[14]^ extended MR to cytokines and blood cell traits, yet few studies have directly addressed the effect of neutrophil count on the risk of lung cancer development and prognosis. To our knowledge, no MR study has specifically examined genetically predicted neutrophil count in relation to lung cancer outcomes. Thus, our study fills this gap by combining MR and observational survival analysis. Additionally, survival analysis offers insights into the utility of specific biomarkers for predicting prognosis in patients with lung cancer. Therefore, combining MR analysis with survival analysis offers a novel approach to evaluating the causal relationship and clinical significance of the neutrophil count in lung cancer.

This research used data from the National Health and Nutrition Examination Survey (NHANES) and the Medical Research Council Integrative Epidemiology Unit OpenGWAS databases. The data were analyzed using both survival analysis and MR analysis to investigate the causal relationship between neutrophil count and the risk of incident lung cancer and explore the effect of neutrophil count on survival in individuals with lung cancer.

## 2. Materials and methods

### 2.1. MR analysis

#### 2.1.1. Data sources

The MR analysis was conducted using aggregated data of genome-wide association studies (GWASs) cohorts of men and women of European descent from studies by Astle et al^[15]^ and Wang et al^[16]^ (Table 1).

**Table 1:** Details of the genome-wide association study data used in the Mendelian randomization analysis.

Phenotype	First author (reference)	Number of cases	Number of controls	Sample size	Number of variants	Racial origin	Year	Trait ID in GWAS
Exposure								
Neutrophil count	Astle^[15]^	NA	NA	170,702	29,164,935	European	2016	ebi-a-GCST004629
Outcome								
Lung cancer	Wang^[16]^	11,348	15,861	27,209	8,945,893	European	2014	ieu-a-966
Squamous cell lung cancer	Wang^[16]^	3275	15,038	18,313	8,893,750	European	2014	ieu-a-967
Lung adenocarcinoma	Wang^[16]^	3442	14,894	18,336	8,881,354	European	2014	ieu-a-965

GWAS = genome-wide association study.

The single-nucleotide polymorphisms (SNPs) used to assess the causal relationship between neutrophil count and lung cancer were obtained from a large-scale study of 11,348 individuals of European descent and 15,861 controls, including 3442 with lung adenocarcinoma and 3275 with squamous cell lung cancer.

#### 2.1.2. Selection of IVs

To identify SNPs significantly associated with neutrophil count from a publicly available GWAS database, the IVs were required to meet the association, independence, and exclusion assumptions to ensure the validity of the analysis results. MR methods rely on 3 key assumptions for selecting IVs: the genetic variant should show a significant association with the exposure of interest; it must be closely linked to the exposure of interest and not affected by any confounding factors; and it should be independent of the outcome, considering both the exposure and all confounding factors. The following steps were used to select appropriate IVs related to neutrophil count from the GWAS results for MR analysis: SNPs that reached the genome-wide significance level (*P* < 5 × 10^−8^) were chosen; the linkage disequilibrium threshold was set at *r*^2^ > 0.001 within a 10,000 kb window, and palindromic SNPs with moderate allele frequencies were excluded from the analysis; when reconciling exposure and outcome data, confounding factors were removed using the National Institutes of Health LDlink tool (https://ldlink.nih.gov/)^[17]^; and the *F*-statistic was used to assess the strength of the association between IVs and the exposure. The *F*-statistic of SNPs was computed to assess potential bias from weak IVs. IVs with an *F* > 100, indicating a strong tool, were selected to minimize the risk of weak IV bias.

#### 2.1.3. Statistical analysis of the MR data

In this study, we aimed to investigate the potential causal relationship between neutrophil count and the incidence of lung cancer, lung adenocarcinoma, and squamous cell lung cancer, treating neutrophil count as the exposure variable and each cancer type as the outcome variable. Multiple methods, including inverse-variance weighted regression, weighted median, and MR-Egger regression, were used to assess the magnitude of the effect on the association between exposure and outcome and investigate the impact of exposure on the outcomes. Inverse-variance weighting (IVW) served as the primary method for MR analysis, whereas Cochrane’s *Q* values, estimated by IVW and MR-Egger regression, were used to assess the heterogeneity among the chosen IVs. The MR-Egger regression intercept was used to assess the horizontal pleiotropy of the chosen IVs. To assess the robustness of the findings, sensitivity analyses – including leave-one-out and MR-intercept methods – were conducted to identify potential biases, limitations, or uncertainties in the MR analysis. The leave-one-out approach was applied by sequentially removing each SNP to evaluate the impact on the inferred causal relationship.

### 2.2. Analysis of the NHANES data

NHANES is a continuous national survey conducted by the US National Center for Health Statistics to evaluate the health and nutritional status of both adults and children in the United States. Data on lung cancer cases identified among NHANES participants between 1999 and 2018 were analyzed to explore the relationship between neutrophil count and lung cancer prognosis. Survival analysis was performed to evaluate the impact of neutrophil count on lung cancer outcomes. In the multivariable Cox regression analyses, we adjusted for key demographic and clinical covariates, including age, sex, body mass index (BMI), smoking status, hypertension, and diabetes. These variables were selected based on their established associations with lung cancer prognosis and their availability in the NHANES dataset. Stratified survival analyses were performed to investigate whether age modified the association between neutrophil count and prognosis. Patients were categorized into 2 age groups (<65 years and ≥65 years). Neutrophil counts were dichotomized into high and low groups according to whether they were above or below the median value, respectively. Kaplan–Meier survival curves were generated separately for each age group, and stratified Cox proportional hazards models were fitted to estimate hazard ratios (HRs) and 95% confidence intervals (CIs).

### 2.3. Statistical analysis

All statistical analyses were conducted using R version 4.5.1 (The R Foundation for Statistical Computing, Vienna, Austria). The 2-sample MR package “TwosampleMR” was used for the MR analysis. Odds ratios (ORs) and HRs with corresponding 95% CIs were calculated using logistic regression and Cox regression, respectively. Statistical significance was defined as a 2-tailed *P* value <.05. Statistical power was assessed using the R package pwr (*f*^2^ = 0.15, α = 0.05), yielding a power of 0.86 for the Cox regression model with 5 predictors, suggesting adequate sample size. Sensitivity analyses confirmed the stability of the results.

As all analyses were conducted using publicly available, fully de-identified data, no ethical approval was required.

## 3. Results

### 3.1. MR analysis

#### 3.1.1. SNP selection

Table 2 presents the findings of the MR analyses with neutrophil count as the exposure variable and lung cancer, lung adenocarcinoma, and squamous cell lung cancer as the outcome variables.

**Table 2:** Mendelian randomization estimation of the stochastic relationship between neutrophil count and risk of developing lung cancer.

Outcomes	nSNPs	IVW method		Weighted median		MR-Egger		Cochran’s *Q*	Egger intercept *P*
		OR (95% CI)	*P*	OR (95% CI)	*P*	OR (95% CI)	*P*		
Lung cancer	11	0.77 (0.62–0.95)	**.017**	0.69 (0.51–0.94)	**.0173**	0.64 (0.31–1.32)	.261	0.401	.629
Lung adenocarcinoma	11	0.63 (0.45–0.87)	**.005**	0.61 (0.83–0.96)	**.033**	0.50 (0.18–1.41)	.224	0.831	.627
Squamous cell lung cancer	11	0.88 (0.61–1.26)	.476	0.84 (0.53–1.32)	.455	0.60 (0.18–1.97)	.422	0.527	.274

Bold values indicates are statistically significant.

CI = confidence interval, IVW = inverse-variance weighted, MR = Mendelian randomization, nSNPs = nonsynonymous single-nucleotide polymorphisms, OR = odds ratio.

Eleven IVs were significant at the genome level, with *F* > 100. Sensitivity analysis confirmed that the initial MR results complied with the 3 MR assumptions. First, we identified 11 SNPs associated with neutrophil count and lung cancer at significant levels (*P* < 5.0 × 10^−8^) after deleting disqualified genes (Table 3). Second, the leave-one-out analysis indicated that systematically removing each SNP did not alter the overall effect on lung cancer and lung adenocarcinoma. The MR-Egger regression analysis showed no evidence of significant horizontal pleiotropy, confirming that the 2nd MR assumption was satisfied. Furthermore, we used the National Institutes of Health LDlink tool to control for the confounding effect of smoking on lung cancer outcomes, ensuring that the 3rd assumption was not violated. Therefore, sensitivity analysis confirmed that the 11 neutrophil count-associated nonsynonymous SNPs satisfied the 3 basic assumptions of strong IVs. Additional sensitivity analyses using mendelian randomization pleiotropy residual sum and outlier and weighted mode were attempted but did not yield stable results owing to the limited number of SNPs (n = 11), consistent with previous reports that the performance of mendelian randomization pleiotropy residual sum and outlier is suboptimal with fewer than 20 instruments. Therefore, we relied on IVW, MR-Egger, and weighted median estimates, all of which showed consistent effect directions.

**Table 3:** SNP associated with neutrophil count and lung cancer at significant levels (*P* < 5.0 × 10^−8^) after deleting disqualified genes.

SNP	Effect allele	Beta coefficient	Standard error	*P* value	*F*-statistic
rs11725704	G	0.0727863	0.00369598	2.46E−86	425.5534444
rs2082382	A	−0.0361828	0.00363451	2.39E−23	110.8369465
rs445	T	−0.0979808	0.0060661	1.1E−58	287.8299893
rs2208568	C	−0.0652968	0.00581445	2.9E−29	140.4579667
rs16850073	T	0.0533551	0.00371506	8.98E−47	228.0658131
rs12266014	T	−0.0440701	0.00371827	2.09E−32	154.473273
rs4760	G	−0.0861491	0.00492275	1.43E−68	335.830874
rs10882895	G	0.046974	0.00364864	6.27E−38	181.5073164
rs791357	C	−0.0414957	0.0040541	1.37E−24	118.4900645
rs2038700	C	0.0353863	0.00367608	6.2E−22	102.0775449
rs56388170	T	0.0749767	0.00398018	3.72E−79	400.708576

SNP = single-nucleotide polymorphism.

#### 3.1.2. Effect of neutrophil count on overall lung cancer incidence

**Figure 1.**
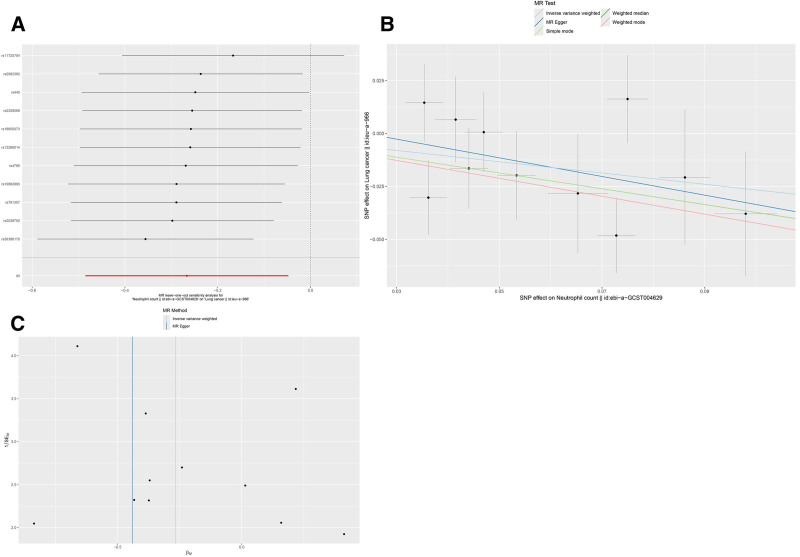
Mendelian randomization (MR) analyses for neutrophil count and lung cancer. (A) Leave-one-out sensitivity analysis; (B) scatter plot; (C) funnel plot. SNP = single-nucleotide polymorphism.

The IVW analysis revealed that higher neutrophil counts were associated with a decreased risk of lung cancer (OR: 0.77, 95% CI: 0.62–0.95, *P* = .017). The analysis showed no evidence of horizontal pleiotropy (*P* = .629) or heterogeneity (*P* = .401), as shown in Table 2. The leave-one-out analysis confirmed the stability of the results (Fig. 1A).

The beta values obtained from the MR-Egger regression and weighted median analyses were consistent with the overall findings. The scatter plot (Fig. 1B) and funnel plot (Fig. 1C) both demonstrated an inverse causal link between neutrophil count and lung cancer risk, with higher neutrophil counts being associated with a lower risk of developing lung cancer.

#### 3.1.3. Effect of neutrophil count on lung adenocarcinoma incidence

**Figure 2.**
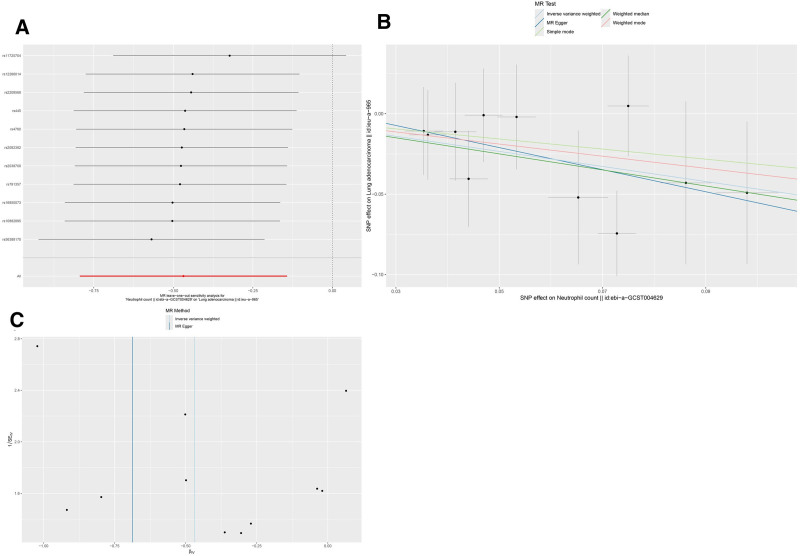
Mendelian randomization (MR) analyses for neutrophil count and lung adenocarcinoma. (A) Leave-one-out sensitivity analysis; (B) scatter plot; (C) funnel plot. SNP = single-nucleotide polymorphism.

According to the IVW analysis, increased neutrophil counts were significantly associated with a decreased likelihood of developing lung adenocarcinoma (OR: 0.63, 95% CI: 0.45–0.87, *P* = .005). The analysis showed no evidence of horizontal pleiotropy (*P* = .672) or heterogeneity (*P* = .831), as shown in Table 2. The leave-one-out analysis confirmed the stability of the results (Fig. 2A). The beta values obtained using MR-Egger regression and weighted median methods aligned with the overall findings. The scatter plot (Fig. 2B) and funnel plot (Fig. 2C) both demonstrated an inverse causal link between neutrophil count and lung adenocarcinoma risk, with higher neutrophil counts being associated with a lower risk of developing lung adenocarcinoma.

#### 3.1.4. Effect of neutrophil count on squamous cell lung cancer incidence

**Figure 3.**
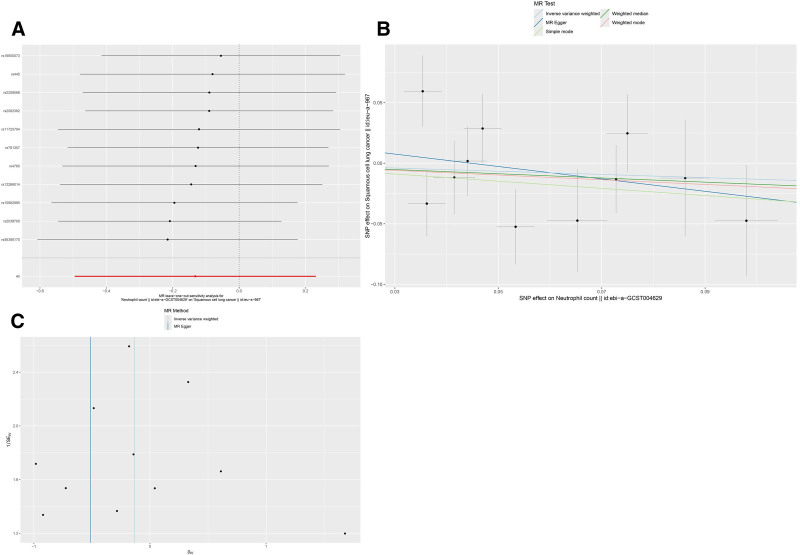
Mendelian randomization (MR) analyses for neutrophil count and lung squamous cell cancer. (A) Leave-one-out sensitivity analysis; (B) scatter plot; (C) funnel plot. SNP = single-nucleotide polymorphism.

The MR analysis did not reveal an association between neutrophil count as the exposure and squamous cell lung cancer as the outcome (OR: 0.88, 95% CI: 0.61–1.26, *P* = .476). The analysis showed no evidence of horizontal pleiotropy (*P* = .527) or heterogeneity (*P* = .274), as shown in Table 2. The leave-one-out analysis also did not show evidence of a causal link between neutrophil count and squamous cell lung cancer (Fig. 3A). The beta estimates obtained from the MR-Egger regression and weighted median analyses were consistent with a lack of causal relationship between neutrophil count and squamous cell lung cancer. The scatter plot (Fig. 3B) and funnel plot (Fig. 3C) both showed a lack of causal relationship between neutrophil count and squamous cell lung cancer.

### 3.2. Survival analysis of the NHANES data

**Figure 4.**
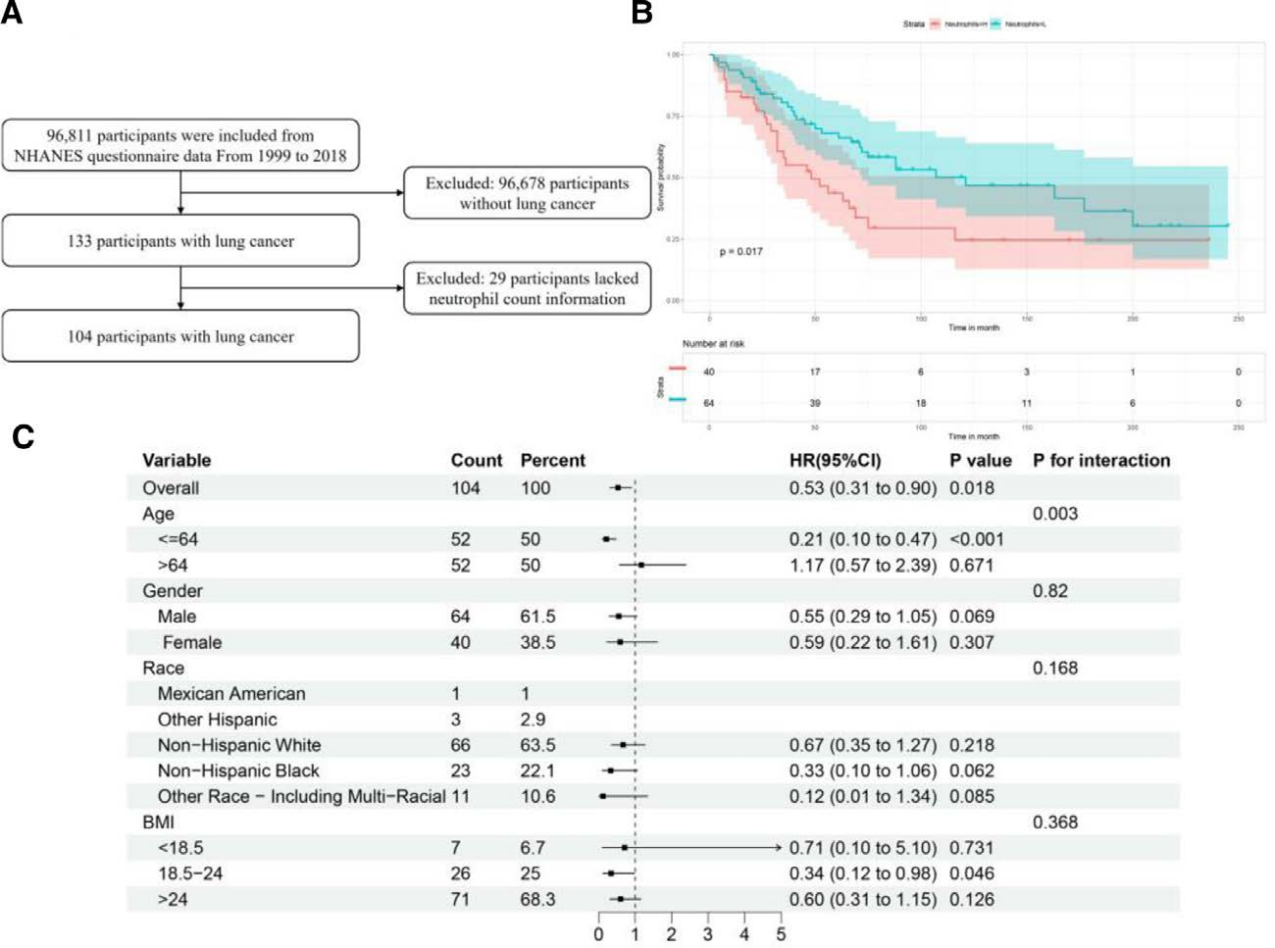
Effect of neutrophil count on survival in adults with lung cancer from the NHANES database. (A) Process for selecting adults with lung cancer; (B) Kaplan–Meier survival curve of adults with lung cancer; (C) results of subgroup analyses to determine the impact of potential risk factor interactions on the association between neutrophil count and survival. Adjusted for age, sex, BMI, smoking status, hypertension, and diabetes. BMI = body mass index, CI = confidence interval, HR = hazard ratio, NHANES = National Health and Nutrition Examination Survey.

A total of 134 adults with lung cancer who participated in NHANES between 1999 and 2018 (Fig. 4A) were identified. However, 29 of the 134 individuals did not have information on their neutrophil count and were, therefore, excluded from the analysis, leaving 104 participants in the analysis (Fig. 4A). Survival analysis showed that a lower neutrophil count was associated with longer survival in individuals with lung cancer (HR: 0.53, 95% CI: 0.31–0.90, *P* = .017; Fig. 4B). Figure 4C presents the results of the subgroup analyses of survival in individuals with lung cancer, stratified by age, sex, race, and BMI.

There was a significant interaction between a high neutrophil count and age (*P* = .003): a high neutrophil count had a significant effect on prognosis in adults under 65 years (HR: 0.21, 95% CI: 0.10–0.47, *P* < .001) but was not associated with prognosis in those aged 65 years and older (HR: 1.17, 95% CI: 0.57–2.39, *P* = .671). There were no significant interactions between neutrophil count with sex, race, or BMI and survival in adults with lung cancer (*P* values for interaction all >.05).

**Figure 5.**
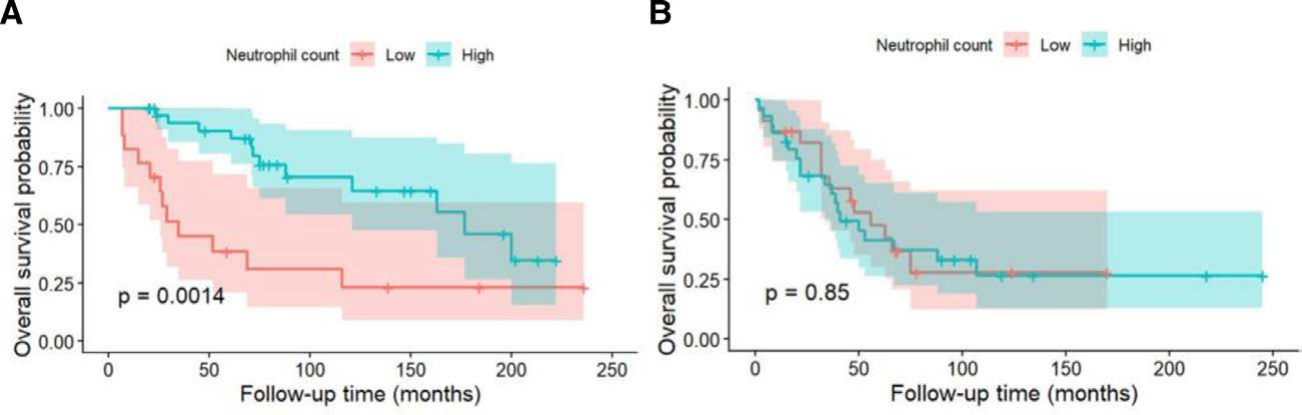
Kaplan–Meier curves stratified by neutrophil count in different age groups. (A) Among patients aged <65 years, higher neutrophil count was significantly associated with worse survival (log-rank *P* = .0014). (B) No significant difference was observed in patients aged ≥65 years (log-rank *P* = .85). Shaded regions represent 95% confidence intervals.

In age-stratified survival analyses, a high neutrophil count was significantly associated with poorer overall survival in patients aged <65 years (HR: 3.47, 95% CI: 1.54–7.78, *P* = .003; log-rank *P* = .0014). In contrast, a high neutrophil count was not significantly associated with overall survival in those aged ≥65 years (HR: 1.06, 95% CI: 0.53–2.13, *P* = .87; log-rank *P* = .85). These findings were consistent across different survival metrics, suggesting that the prognostic role of neutrophils is confined to younger patients (Fig. 5 and Table 3).

## 4. Discussion

The MR analysis revealed a causal link between elevated neutrophil counts and a reduced risk of lung cancer, including lung adenocarcinoma and squamous cell lung cancer. Our study established a relationship between neutrophil count and lung cancer risk and offered a novel approach for identifying individuals more likely to develop lung cancer. Moreover, the data from the NHANES database revealed the complex role of neutrophil count in lung cancer prognosis and the modifying effect of age.

Neutrophils are a vital part of the innate immune system and constitute the largest white blood cell fraction. They are key defenders against infection.^[18]^ However, recent studies have revealed that neutrophils are also involved in the onset and progression of cancer, including lung cancer, in a complex and varied manner.^[6,19]^ As an important part of the TME, neutrophils participate in the inflammatory regulation, angiogenesis, and immune escape of tumors by secreting cytokines and chemokines and forming neutrophil extracellular traps.^[20,21]^ These findings have made neutrophils a focus of interest in cancer research and treatment.

Exhibiting dual roles in tumor progression, neutrophils either promote or inhibit tumor growth depending on the tumor stage and TME.^[22]^ They facilitate tumor invasion, metastasis, and angiogenesis by releasing matrix metalloproteinases, vascular endothelial growth factor, and pro-inflammatory factors such as interleukin-6 and CXCL8, which degrade the extracellular matrix and recruit immunosuppressive cells. Neutrophils also form neutrophil extracellular traps, which enhance metastasis by trapping circulating tumor cells and degrading the extracellular matrix through proteases such as elastase.^[23–25]^ Conversely, tumor-associated neutrophils can adopt an N1 phenotype early on, promoting antitumor immunity, but often shift to a pro-tumor N2 phenotype as the tumor progresses.^[26,27]^ Additionally, neutrophils suppress T-cell activity and promote immune escape via reactive oxygen species, nitrogen oxides, and immune checkpoint molecules such as programmed cell death ligand 1, highlighting their complex and stage-dependent roles in cancer.^[19]^

Our study showed a significant interaction between neutrophil count and age in adults with lung cancer: a high neutrophil count had a significant effect on prognosis in adults aged under 65 years, but was not associated with prognosis in adults aged 65 years and older. Consistent with this age-dependent pattern, our stratified analyses showed that elevated neutrophil count predicted significantly worse survival in patients aged <65 years but was not associated with survival among those aged ≥65 years. This finding has not been reported in previous studies. Jackaman et al^[28]^ found that accumulation and functional impairment of neutrophils are common among older adults. This phenomenon may be attributed to a reduction in neutrophil apoptosis in older adults. In older adults, resting neutrophils exhibit a pre-activated basal state, which promotes inflammation. However, under inflammatory stimuli, the response of neutrophils may be less effective, thereby weakening the immune response. Although activated neutrophils retain their adhesion capacity, their chemotactic response to various stimuli is significantly diminished. This leads to unclear migration patterns of neutrophils in response to stimuli. Additionally, the phagocytic activity of neutrophils is reduced in older adults. After phagocytosis, the production of reactive oxygen species and the release of cytotoxic proteases from neutrophil granules are also reduced. These phenomena collectively result in a reduction in neutrophil cytotoxic activity and an impaired immune response in older adults.

Inflammatory markers associated with neutrophils are important for diagnosing lung cancer, predicting prognosis, and assessing treatment response. The neutrophil-to-lymphocyte ratio is one of the most widely used neutrophil-related indicators.^[29]^ A higher neutrophil-to-lymphocyte ratio is associated with better outcomes in patients with lung cancer.^[30]^ This may be because higher neutrophil counts reflect a stronger inflammatory response, whereas lower lymphocyte counts suggest suppression of immune function.

Our analysis revealed a causal relationship between neutrophil count and the overall risk of lung cancer. However, further analysis by type of lung cancer revealed that increased neutrophil counts were associated with a decreased risk of lung cancer and lung adenocarcinoma, but no causal relationship was found between neutrophil count and squamous cell lung cancer. In addition, Kaplan–Meier survival analysis showed that a lower neutrophil count was associated with significantly longer overall survival in individuals with lung cancer than a higher neutrophil count. Cox regression revealed a significant interaction between a high neutrophil count and age, and the effect of a high neutrophil count on prognosis was significant in adults aged under 65 years. The MR analysis and Kaplan–Meier survival analysis had consistent results. Neutrophil count, as a routine blood test indicator, has the advantages of being an inexpensive and convenient test and could be used for prognostic risk assessment in younger patients (aged under 65 years) with lung cancer and included in a lung cancer prognosis prediction model.^[28,31]^

The apparent opposite directions of association observed in our study – protective effects in the MR analysis but adverse effects in the survival analysis – may be explained by several biological and methodological factors. First, MR analysis estimates the lifelong effect of genetically determined neutrophil levels in cancer-free individuals, reflecting baseline immune activity before disease onset. In contrast, the survival analysis captures the impact of neutrophil levels after tumor development, which are often influenced by tumor burden, inflammation, and treatment-related factors. Elevated circulating neutrophil counts in patients with lung cancer may reflect a tumor-promoting inflammatory state rather than a causal protective effect. Second, the dual nature of neutrophils within the TME might explain the discrepancy: neutrophils can exhibit both antitumor (N1) and pro-tumor (N2) phenotypes depending on cytokine signaling, disease stage, and microenvironmental cues. Thus, genetically higher neutrophil levels may enhance early immune surveillance, whereas elevated neutrophil counts in patients with cancer could facilitate tumor progression. Third, the possibility of reverse causation in the observational survival data cannot be ruled out, as systemic inflammation and tumor progression can cause elevated neutrophil counts. Collectively, these results suggest that the role of neutrophils in lung cancer is context-dependent: they are potentially protective during initiation but detrimental during progression.

The study has some limitations. First, although MR analysis can reduce confounding, the choice of IVs depends on GWAS data and may be limited by genetic diversity and statistical power, and other confounding factors, including environmental confounding factors, may affect the MR analysis results. Second, because the GWAS study included only participants of European descent, the findings may not be applicable to individuals with other racial backgrounds. Third, this study concentrated on the causal relationship between neutrophil count and lung cancer, and further research is needed to investigate the possibility of reverse causality. Finally, owing to the retrospective nature of this study, which relied on existing data, we were unable to investigate the effects of dynamic changes in inflammation-related biomarkers on lung cancer.

## 5. Conclusions

This study strengthens the evidence linking neutrophil counts to lung cancer, with neutrophils acting as a crucial component of the TME and playing a complex role in preventing both the initiation and progression of the disease. The findings suggest that neutrophil count could serve as a valuable biomarker for assessing lung cancer risk. Future studies should investigate the mechanisms through which neutrophils affect the onset and progression of lung cancer, and the potential role of neutrophils in predicting prognosis and treatment monitoring. Additionally, the specific pathways through which neutrophils affect lung cancer development and outcomes should be explored.

## Acknowledgments

The authors thank the investigators and participants who contributed to the GWAS data and the NHANES data used in this study. The datasets generated and/or analyzed during the current study are available in the published GWASs and NHANES database (https://www.cdc.gov/nchs/nhanes).

## Author contributions

**Conceptualization:** Shuai Sun.

**Data curation:** Qinghai Meng, Huan Wang, Xiaoren Hu, Binbin Lan, Rui Liu, Hanqing Li.

**Writing – original draft:** Chunpeng Meng, Shuai Sun.

**Writing – review & editing:** Chunpeng Meng, Zhifeng Zheng, Jianhua Li, Yong Liu, Shuai Sun.
